# Nutritional portrait of processed foods purchased in Québec (Canada), 2016–2022

**DOI:** 10.1017/S1368980025000588

**Published:** 2025-04-21

**Authors:** Julie Perron, Alicia Corriveau, Sonia Pomerleau, Clara-Jane Rhéaume, Marie-Ève Labonté, Véronique Provencher

**Affiliations:** 1 Centre NUTRISS - Nutrition, Santé et Société, Institut sur la nutrition et les aliments fonctionnels (INAF), Université Laval, Québec, QC, Canada; 2 École de Nutrition, Université Laval, Quebec, QC, Canada

**Keywords:** Food supply, Nutritional value, Food purchases, Front-of-pack labelling

## Abstract

**Objective:**

The Food Quality Observatory synthetises the nutritional composition of fifteen processed food categories commonly purchased in Québec (Canada). We assessed how the new Canadian front-of-pack (FoP) labelling regulation of a ‘high in’ symbol, to be implemented as of January 1, 2026, would be potentially reflected in these categories and how simulations of reformulation would impact the presence of the symbol.

**Design:**

Nutritional information was obtained by collecting food products available in supermarkets and grocery stores in the province of Québec (2016–2022). Sales data were obtained from *NielsenIQ* company. Fifteen food categories have been selected, and three levels of reformulation were simulated.

**Setting:**

The nutritional values of 5132 products were merged with sales data. 3941 products were successfully cross-referenced.

**Results:**

Sixty percent of all products sold (*n* 2336/3941) would carry the ‘high in’ symbol reflecting a high content of Na, saturated fat and/or total sugar (39 %, 16 % and 17 %, respectively). For certain food categories, a slight reduction (5–15 %) in Na, saturated fat or total sugar content would allow removing the ‘high in’ symbol in a large number of products. For example, a 5 % reduction of the Na content in sliced breads would allow 22 percentage point (pp) fewer products to display the symbol.

**Conclusions:**

This study presents a portrait of processed foods purchased in Québec (Canada) and the distribution of the FOP ‘high in’ symbol. Such a portrait generates important data to monitor the food supply’s nutritional quality, which can ultimately contribute to improving the nutritional quality of processed foods.

## Graphical abstract

**Figure:**
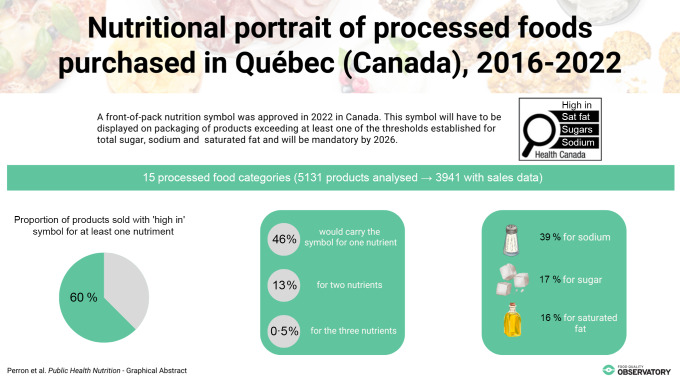

A food supply that offers products with high levels of Na, saturated fat and/or sugar is considered a major cause of non-communicable diseases^(1)^. Several strategies have been implemented to facilitate consumers’ choices towards more nutritious food and improve the food supply’s nutritional quality. Among these strategies, front-of-pack (FoP) labelling has been identified as effective in promoting healthy food environments and helping consumers make healthier food choices^(2,3)^. In 2016, Chile adopted a FoP warning symbol for food products exceeding specified amounts of energy, saturated fat, sugar or Na^(4)^. Since then, Peru, Mexico, Israel, Brazil, Venezuela, Uruguay, Argentina and Colombia have enacted policies requiring FoP warning labels similar to Chile’s^(5,6)^. A comparable approach has been recently adopted in Canada with a FoP nutrition symbol approved in 2022, which will be mandatory by 2026^(7)^. This symbol will have to be displayed on the packaging of products exceeding at least one of the thresholds established for total sugar, Na and saturated fat, based on the food category to which they belong. For example, for products with a reference amount of 30 g or less, the threshold is 10 % of the recommended daily value (DV), specific to each nutrient, compared to 15 % of DV for products with a reference amount of more than 30 g.

FoP nutrient warning is increasing across the globe since it has been shown to be easy to understand, not to mention that it is an effective strategy to help consumers identify products high in nutrients to limit and therefore to discourage them from purchasing these products^(5,8,9)^. An underlying aim of such a FoP label is to motivate food industries to reformulate their products to avoid the symbol on their packaging. In 2022, the WHO published a policy brief which supports the reformulation of foods and beverages^(10)^. Data from this report support that consumers accept reformulated products, and that reformulation leads to better nutritional intakes and better health. Reformulation of Na, saturated fat and sugar contents can indeed contribute to ensuring access to more nutritious food and shifting towards healthier consumption patterns. The advantage of food reformulation is that consumers do not need to modulate their buying behavior or make conscious efforts to choose healthier options.

Na is the most reformulated nutrient since sixty-two countries have reformulation strategies to reduce Na in packaged foods^(11)^. As an example, the UK launched a successful voluntary salt reduction programme that has set five levels of progressively more stringent Na targets since 2004^(12,13)^. Seven years later, this programme has led to reductions of up to 45 % in Na levels in some products and has led to a 15 % drop in population salt intakes. However, such a voluntary programme in Canada has not produced the expected results^(14)^. Sugar reformulation strategies are less common. In the UK, a 3·5 % reduction in total sugar of products sold was observed 4 years after the implementation of the voluntary sugar reduction programme in 2016^(15)^. Reformulation to reduce saturated fat by the food industry has been much less studied and has had little success, while WHO suggests that saturated fat be replaced by unsaturated fat instead of reducing saturated fat alone^(10)^.

To our knowledge, very few studies have measured the impact of FoP nutrient symbol strategies on food reformulation,^(16)^ and none of these studies have been conducted in Canada. It is thus of relevance to monitor the nutritional composition of the food supply prior to implementing such a strategy, in order to ultimately measure the impact of the FoP nutrition symbol on consumer choice and industry reformulation of foods. While the nutritional composition of packaged processed foods and beverages in Canada has been previously studied based on products available on shelves^(17,18)^, no study has yet measured the nutritional composition of processed foods that are actually purchased in the country. Therefore, the aims of this study were first to characterise the nutritional composition of fifteen processed food categories commonly purchased in the province of Québec (Canada). We then verified the extent to which the Canadian FoP nutrition symbol would be displayed in those food categories and verified the number of nutrients concerned. Finally, we simulated the effects of various levels of reformulation (5 %, 10 % and 15 % reduction) for Na, saturated fat and total sugar on the distribution of the FoP nutrition symbol.

## Methods

### Data collection

Hosted by the Institute of Nutrition and Functional Foods at *Université Laval*, the Food Quality Observatory (Observatory) was launched in 2016. The Observatory aims at monitoring the evolution of the food supply, hence contributing to the collective effort of improving the quality and accessibility of food. Consultations with the Observatory knowledge users and the scientific committee were conducted to prioritise the processed food categories to monitor (*n* 15)^(19)^. To be retained, the food categories had to have an impact on health (i.e. high in sugar, Na and/or saturated fat), show a variability in terms of nutritional quality, have a high household penetration rate and show a potential for improvement. The following food categories were thus selected for the analyses: ready-to-eat (RTE) breakfast cereals, sliced breads, luncheon meats, ready-to-serve soups, pizzas, granola bars, frozen meals, pasta sauces, yoghurts and dairy desserts, sausages, cookies, crackers, salty snacks, processed cheeses and flavoured milks and plant-based beverages. Note that luncheon meats, yoghurt and dairy desserts, sausages and processed cheeses also included related plant-based alternatives.

To reach the objectives described above, a database containing the nutritional value and labelling information of each product was created by the Observatory (*n* 10 out of fifteen categories), *Protégez-Vous* (*n* 4 out of fifteen categories) – a Québec-based non-profit organisation specialised in consumer information and products testing – and Health Canada (*n* 1 out of fifteen categories). Data collections were done in supermarkets, grocery stores and specialty grocery stores from the Greater Québec City area, the Greater Montreal area (Québec, Canada) or across Canada (for pizzas only) between September 2016 and February 2022. All information on the product packaging (e.g. brand, Nutrition Facts table, list of ingredients, nutrition and health claims, serving size) was coded in the database using double coders. Nutritional value variables listed were as follows: energy (kJ), total fat (g), saturated fat (g), total sugar (g), fibre (g), protein (g) and Na (mg).

This nutritional value database was then merged with a sales database (provided by *NielsenIQ* company^(20)^) by using unique product codes. For each product, the database included the following data: sales in Canadian dollars (CAD$), sales in kilograms (kg) and sales per unit. Sales information comes from the optical reading of the products purchased in the main food chains of Québec markets (or Canada markets for pizzas).

### Statistical analyses

Means and standard deviations of nutritional values and price per serving of products were first generated. The averages were weighted by sales volume in kg to better represent what the population buys – and indirectly consumes – by giving a higher weight to the most popular products of a given food category and a lower weight to the products which were less purchased. Since the analyses weighted for sales were conducted based on the combined database, the number of products analysed was lower than the number of food items found on shelves.

In order to determine if each product would carry the Canadian FoP nutrition symbol, 15 % of the DV threshold was used for most products^(7)^. The 30 % of the DV threshold was used for frozen meals and pizzas since they are considered as mixed dishes. The 10 % of the DV threshold was used for products for which the reference amount was less than or equal to 30 g. The highest amount between the serving size indicated on the package and the reference amount of the product^(21)^ was used for the calculation. Exemptions of the FoP nutrition symbol have also been considered (e.g. yoghurts with high calcium content).

Simulations were performed by modelling different scenarios where food products underwent reformulation to reduce specific components (Na, saturated fat or sugar), by varying percentages of 5 %, 10 % or 15 %. These reductions were then presented for their impact on the distribution of FoP labels. Statistical analyses were conducted using SAS software version 9·4.

## Results

A total of 5132 different products were identified from the fifteen selected food categories. Among them, 3941 products with sales information were successfully cross-referenced with the products identified in the food supply. According to *NielsenIQ* database, the products offered from which sales data were available represented an average market coverage of 79 %.

Table 1 shows the variety of products offered, the number of products with purchases data and the market coverage with *NielsenIQ* database according to each food category, with cookies and salty snacks representing the largest food categories.

**Table 1:** Availability of products among the selected food categories (*n* 15)

Food categories (year of collection)	Availability of products	Products offered matched with sales data	Supply coverage of the *NielsenIQ* database^*^
	Offer (*n*)	Purchases (*n*)	Market coverage (%)
RTE breakfast cereals (2016)	331	308	90
Sliced breads (2016)	294	262	75
Luncheon meats (2017)	361	317	62
Ready-to-serve soups (2017)	223	180	92
Pizzas (2017)	155	155	80
Frozen meals (2018)	386	275	70
Granola bars (2018)	310	240	75
Yogurts and dairy desserts (2018)	380	325	86
Pasta sauces (2019)	322	210	88
Cookies (2019)	694	494	87
Sausages (2019)	289	214	63
Crackers (2020)	439	223	94
Salty snacks (2020)	627	503	91
Processed cheeses (2020)	118	87	72
Flavoured milks and plant-based beverages (2022)	203	148	73
Total	5132	3941	79

*n* = number of unique products per food category; RTE, ready-to-eat.

* Source: Nielsen IQ MarketTrack (2016–2022), Quebec All Channels, 52 weeks.

Table 2 shows the nutritional value of the products purchased (weighted by sales volume) according to each food category. Purchased pizzas, sausages and frozen meals had the highest content in saturated fat per serving. Purchased flavoured milks and plant-based beverages, RTE breakfast cereals and cookies had the highest sugar content per serving. Purchased pizzas, ready-to-serve soups and frozen meals had the highest Na content per serving.

**Table 2:** Mean nutritional value of purchased food categories per portion

	Portion	Calories (kJ)		Lipids (g)		Saturated fat (g)		Carbohydrates (g)		Fibre (g)		Total sugar (g)		Protein (g)		Na (mg)	
		Mean	sd	Mean	sd	Mean	sd	Mean	sd	Mean	sd	Mean	sd	Mean	sd	Mean	sd
RTE breakfast cereals (*n* 308)^*^	55 g	879	84	2·6	2·6	0·8	1·6	44	4	4·4·	3·8	12·8	5·5	4·7	1·7	216	125
Sliced breads (*n* 262)	2 slices (73 g)	791	142	2·3	0·9	0·5	0·2	35	7	2·7·	1·7	3·0	2·4	7·3	1·6	331	83
Luncheon meats (*n* 317)	55 g	414	188	6·2	5·1	2·2	1·8	2	1	0·0·	0·2	0·6	0·7	8·7	2·4	542	148
Ready-to-serve soups (*n* 180)	250 ml	544	213	3·5	4·2	1·1	1·2	19	6	3·9·	3·0	3·5	2·7	5·3	2·6	715	132
Pizzas (*n* 155)	200 g	1983	213	19·5	5·7	7·5	2·5	55	6	3·3·	1·2	6·7	3·0	19·8	3·4	1066	193
Frozen meals (*n* 275)	1 meal (278 g)	1385	418	10·2	6·6	3·7	2·8	44	13	3·1·	1·4	6·8	6·0	15·8	5·4	698	237
Granola bars (*n* 240)	1 bar (34 g)	598	126	5·1	2·4	1·6	1·0	23	5	2·0·	1·3	9·9	3·7	2·5	1·6	94	42
Yogurt and dairy desserts (*n* 325)	1 unit (110 g)	318	121	1·5	1·4	0·9	0·8	12	5	0·1·	0·4	9·2	4·1	3·7	1·5	53	19
Pasta sauces (*n* 210)	125 ml	305	155	3·0	3·7	1·1	2·2	9	2	1·9·	0·8	5·3	1·4	2·5	1·2	476	110
Cookies (*n* 494)	30 g	590	117	5·7	1·7	2·3	1·4	21	5	0·8·	0·7	10·4	3·2	1·6	0·7	87	43
Sausages (*n* 214)	55 g (cooked/pre-cooked) or 75 g (raw)	653	138	12·6	3·5	4·2	1·5	4	1	0·1·	0·3	0·5	0·9	8·2	1·8	483	78
Crackers (*n* 223)	23 g	377	59	3·4	1·4	0·8	0·6	13	2	0·7·	0·7	0·9	1·0	1·7	0·5	150	50
Salty snacks (*n* 503)	50 g	1096	79	14·7	3·7	1·9	1·3	29	4	1·9·	1·0	1·3	1·8	3·2	1·0	332	131
Processed cheeses (*n* 87)	30 g	318	50	5·2	1·7	3·1	1·1	4	2	0·0·	0·1	2·2	1·1	3·8	1·3	365	130
Flavoured milks and plant-based beverages (*n* 148)	250 ml	515	205	3·3	1·1	1·1	0·9	18	10	0·7·	0·8	14·7	10·4	5·3	3·2	149	47

Mean ± sd. RTE, ready-to-eat; FoP, front-of-pack.

* *n* = number of products with sales data.

**Fig. 1:**
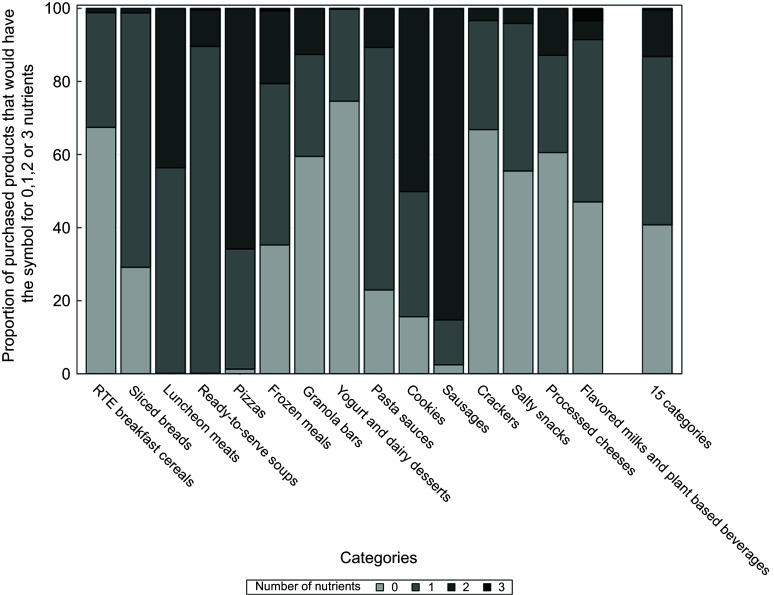
Distribution of requirement to display FoP ‘high-in’ nutrition symbol among purchased food categories. FoP, front-of-pack.

Figure 1 shows the current proportion of products which would carry a FoP nutrition symbol for 0, 1, 2 or 3 nutrients if no changes were made in their food composition. In total, 60 % of products purchased (*n* 2336/3941) would have the FoP nutrition symbol for at least one nutrient. More specifically, 39 % would have the symbol for Na, 17 % for total sugar and 16 % for saturated fat (see Table 3). Almost half of the products purchased (46 %) would carry the symbol for only one nutrient, 13 % for two nutrients and 0·5 % for three nutrients. Yoghurt and dairy desserts, RTE breakfast cereals, crackers and granola bars were the food categories that would most often carry no symbol. On the other hand, sausages, pizzas and cookies were the food categories which would most often carry the symbol for at least two nutrients.

**Table 3:** Distribution of FoP nutrition symbol according to nutrients of interest and simulations of reformulation of 5, 10 and 15 %

	Proportion of products with the FoP nutrition symbol for at least one nutrient (weighted for sales)	Proportion of products with FoP nutrition symbol for Na (weighted for sales)				Proportion of products with FoP nutrition symbol for saturated fat (weighted for sales)				Proportion of products with FoP nutrition symbol for sugar (weighted for sales)			
Food category	Actual	Actual (%)	–5 % (%)^*^	–10 % (%)^†^	–15 % (%)^‡^	Actual	–5 % (%)^*^	–10 % (%)^†^	–15 % (%)^‡^	Actual	–5 % (%)^*^	–10 % (%)^†^	–15 % (%)^‡^
RTE breakfast cereals (*n* 308)	32·6	2·9	0·7	0·2	0·0	7·4	6·8	6·8	6·8	23·4	14·6	8·1	3·8
Sliced breads (*n* 262)	70·8	69·6	47·5	41·5	19·1	0·0	0·0	0·0	0·0	2·4	1·8	1·8	0·0
Luncheon meats (*n* 317)	99·8	99·8	98·3	97·3	96·7	43·6	39·8	39·3	32·6	0·0	0·0	0·0	0·0
Ready-to-serve soups (*n* 180)	99·8	99·8	99·6	99·5	99·0	9·2	5·6	5·6	5·3	1·7	1·7	0·7	0·5
Pizzas (*n* 155)	99·9	93·6	91·6	85·2	79·4	71·0	59·4	52·3	38·1	0·0	0·0	0·0	0·0
Frozen meals (*n* 275)	64·7	63·7	51·0	45·0	33·8	20·1	17·4	16·3	12·3	2·1	1·8	1·5	1·5
Granola bars (*n* 240)	40·5	0·0	0·0	0·0	0·0	22·9	16·9	13·4	7·1	30·2	24·3	20·6	16·3
Yoghurt and dairy desserts (*n* 325)	25·4	0·0	0·0	0·0	0·0	0·5	0·5	0·5	0·4	25·1	24·5	20·8	18·9
Pasta sauces (*n* 210)	77·1	77·1	74·5	64·1	58·4	11·0	10·3	10·3	10·3	0·0	0·0	0·0	0·0
Cookies (*n* 494)	84·4	0·0	0·0	0·0	0·0	57·7	44·9	44·6	44·5	76·7	65·9	49·1	44·9
Sausages (*n* 214)	97·5	97·0	96·8	96·6	92·7	85·7	85·6	85·6	83·3	0·0	0·0	0·0	0·0
Crackers (*n* 223)	33·2	24·5	19·7	7·9	5·5	11·9	10·7	10·7	7·5	0·1	0·0	0·0	0·0
Salty snacks (*n* 503)	44·5	38·4	35·2	29·6	20·7	9·9	6·6	6·6	6·6	0·3	0·3	0·3	0·1
Processed cheeses (*n* 87)	39·5	12·8	11·9	11·9	11·9	39·5	39·5	39·5	39·5	0·0	0·0	0·0	0·0
Flavoured milks and plant-based beverages (*n* 148)	52·9	3·3	0·0	0·0	0·0	11·3	9·8	9·8	0·9	50·2	48·3	48·3	48·3
Total	59·2	39·1	32·4	29·5	22·6	16·3	14·3	13·9	11·8	17·4	15·5	13·4	12·0

RTE, ready-to-eat; FoP, front-of-pack.

* Proportion of products with FoP nutrition symbol with –5 % reformulation.

† Proportion of products with FoP nutrition symbol with –10 % reformulation.

‡ Proportion of products with FoP nutrition symbol with –15 % reformulation.

Table 3 simulates the effects on the distribution of the FoP nutrition symbol of potential reductions (5 %, 10 % or 15 %) in the Na, saturated fat and total sugar content in each food category. Among salient results, a theoretical improvement of only 5 % in Na content of sliced breads would lead to a 22-percentage point (pp) reduction, going from 70 % to 48 %, of products with the FoP nutrition symbol for Na. Moreover, a theoretical improvement of only 5 % in the saturated fat content of pizzas would lead to a 12-pp reduction, going from 71 % to 59 %, of products with the FoP nutrition symbol for this nutrient. Finally, a theoretical reduction of 10 % in total sugar in cookies would lead to a 28-pp reduction, going from 77 % to 49 %, of products with the symbol for sugar. On the other hand, a 15 % reduction in Na content for ready-to-serve soups and luncheon meats or a 15 % reduction in saturated fat for sausages would not be enough to modify the FoP distribution in those food categories.

## Discussion

This novel study provides an overview of the nutritional composition, distribution of potential FoP nutrition symbols and reformulation simulations of processed foods purchased in the province of Québec (Canada). Few studies have examined different food categories simultaneously, and those that have done so have not included weighted sales data in their analyses due to limited access.

### Nutritional composition

First, results showed that the vast majority of food categories analysed in this study have a high Na content. Per 100 g, processed cheeses, luncheon meats and sausages were the categories with the highest Na content, an observation similar to what has been reported in the literature. Indeed, according to another Canadian study carried out on 7234 food items from thirteen food categories, cheese products offered had indeed the highest average Na content (1471 mg/100 g), followed by packaged deli meats (1092 mg/100 g)^(22)^. In Argentina, an analysis conducted on 1320 products from fourteen food categories has revealed that sausages (1050 mg/100g) and ready meals offered (941 mg/100 g) were among the food categories with the highest Na content^(23)^.

Moreover, our study revealed that, per 100 g, purchased cookies, granola bars and RTE breakfast cereals were the categories with the highest sugar content. Similarly, a Canadian study showed that cookies (32 g/100 g), granola bars (30 g/100 g) and RTE breakfast cereals (21 g/100 g) offered had the highest sugar content^(24)^. In the New Zealand food supply, the granola bar category ranked third in terms of sugar content, following jams and confectionery^(25)^.

In this study, processed cheeses, cookies and sausages were among the food categories with the highest content of saturated fat, again per 100g. Although fewer studies have been carried out on saturated fat, a New Zealand study reported that the highest saturated fat content was in cheeses (18 g/100 g), biscuits (8 g/100 g) and processed meats (6 g/100 g)^(25)^.

In summary, food categories high in Na, sugar or saturated fat are similar elsewhere in Canada and around the world. Moreover, for all nutrients of concern, a large variability was observed between food categories and within each category (e.g. Na content varies from 112 mg to 373 mg/100 g in ready-to-serve soups containing vegetables, starch and proteins^(26)^, sugar content varies between 3 g and 15 g/100 g for stirred yoghurts^(27)^ and saturated fat content varies between 2 g and 8 g/100 g for deli meat pizzas)^(28)^. Such findings suggest that a reduction is possible and realistic for a large proportion of products.

### FoP nutrition symbol

In total, unless any changes are made in the nutritional composition, more than half of the products purchased and analysed in this study would carry the Canadian FoP nutrition symbol for at least one nutrient, with Na being the most common nutrient displayed. Accordingly, a research team in the province of Ontario (Canada) reported that a total of 66 % of the food offered on shelves would carry the FoP nutrition symbol (32 % for Na, 28 % for sugar and 28 % for saturated fat)^(17)^. However, the comparison with our study is limited, as it did not incorporate weighted sales data, and it covered a wider range of food categories. In 2019 in Chile, at the beginning of the FoP warning implementation (final stage), 83 % of nearly 5500 food products had the symbol for at least one nutrient^(29)^.

In the current study, one product out of eight would have the FoP nutrition symbol for two nutrients, while a minority of products (0·5 %) would have it for all nutrients. In comparison, in another Canadian study, the majority of consumed foods that would display a FoP nutrition symbol would be for only one nutrient accounting for 18 % of energy intakes^(30)^. Foods that would display a FoP symbol for two and three nutrients accounted for 6 % and 0·1 % of food energy intakes, respectively. This study demonstrated that luncheon meats, ready-to-serve soups and pizzas were the categories which would carry the FoP nutrition symbol for almost all products purchased. In Chile, sweet baked products, soups and savoury snacks were the food categories with the most prevalent warning symbol before the implementation of the regulation (100 %, 98 % and 94 %, respectively)^(31)^.

### Simulations of reformulation

This study demonstrates that reductions in the order of 5, 10 or 15 % in Na, saturated fat or total sugar can have a major impact on products with a potential Health Canada FoP nutrition symbol, with a large proportion of them being able to avoid such a symbol. Considering that reformulated products without nutritional warnings were perceived in a Chilean study as healthier and had higher purchase intention scores than their regular counterparts with a warning^(32)^, this can be a motivating call to action for food industries. Since those food categories are largely consumed by Canadians, these little changes can reduce purchases of nutrients of public health concern and ultimately reduce their intake in the population. Modelling studies have shown that modest reductions, if applied to all products of most purchased food categories, have the potential to improve population health^(33–35)^. As observed above, Na is the nutrient for which reformulations should be attempted in priority. In a systematic review, Na reformulation was carried out more often in breads, sauces and processed meats^(35)^. In Chile, the most frequent reductions of FoP prevalence in ‘high in sodium’ concerned savory spreads, cheeses, RTE meals, soups and sausages^(31)^.

However, this does not dismiss the fact that reformulation is challenging. Numerous considerations are raised on technical feasibility, shelf life, palatability and food safety, which can be discouraging for the food industry^(36,37)^. Moreover, some adverse effects of the FoP warning symbol and reformulation may occur. Indeed, some countries noted an increase in the use of non-nutritive sweeteners as a replacement for sugar to avoid the warning symbol^(38,39)^. Since non-nutritive sweeteners have been associated with adverse metabolic effects, particularly in youth (e.g. altered sweetness perceptions, gut microbiota dysbiosis and disruption of glucose homoeostasis)^(40–42)^, some countries now display a warning on products for the presence of non-nutritive sweeteners^(43)^. Similarly, the WHO discourages the use of non-nutritive sweeteners in place of sugar^(44)^. As another collateral effect, some stakeholders anticipate the introduction of additives in recipes to avoid the FoP (e.g. potassium salts or monosodium glutamate to compensate for the diminution of Na). The long-term effects of these additives on health are not yet known.

This study has several strengths. While access to sales data is expensive, the combination of nutritional composition with sales data to monitor the actual foods purchased is an important strength of this study. Another strength is that the sales data cover a large proportion of the food supply for all food categories studied, with an average of approximately 80 % overall. Moreover, this study is among the first to propose simulations of reformulation specific to each food category. However, this study also has some limitations. Although the fifteen food categories identified are widely bought by consumers, they do not cover all processed food categories. Also, since data collection and analysis of a single food category lasted approximately 9 months, it was not possible to monitor all fifteen food categories during the same year. Furthermore, the nutritional database is an overview at a given time, which may not represent the whole portrait of the food supply during the entire year and does not evolve in real time. Different products may not have been identified, such as products that entered the market after data collection or those purchased at another moment during the year but that were discontinued before the data collection. Additionally, not all products were successfully matched to sales data. In fact, the sales database available through the *NielsenIQ* company does not include products from certain private labels of specific grocery stores. Moreover, even if food sales data can give an overview of food intakes^(45,46)^, it is not possible to ensure that the products purchased are truly eaten by the consumers who bought them.

In conclusion, this study presents an initial portrait of fifteen food categories highly consumed in Québec (Canada), which represents the starting point for monitoring the nutritional composition of food purchased in the province. In accordance with current food-related policies, monitoring the nutritional composition of food purchased after the implementation of the FoP Canadian regulation will be essential over the next few years^(47)^. In that context, the Observatory will continue to monitor the composition of processed food purchases every 5 years. Based on these findings, it becomes possible to identify areas of improvement regarding the nutritional composition of processed foods, which is of great relevance for policymakers and public health nutrition advocates with the aim to encourage healthier food choices.

The full report is freely available on www.observatoire.inaf.ulaval.ca.

## Supporting information

Perron et al. supplementary material Perron et al. supplementary material

## Supplementary material

For supplementary material accompanying this paper visit https://doi.org/10.1017/S1368980025000588
