# Supplementary material for: Nutritional portrait of processed foods purchased in Québec (Canada), 2016–2022
Source: Public Health Nutr. 2025 Apr 21;28(1):e79. doi: 10.1017/S1368980025000588 (PMC12171924; doi:10.1017/S1368980025000588)
Supplement: Perron et al. supplementary material [file S1368980025000588sup001.docx]

**Supplementary Table 1. Thresholds to determine if a product would display the FoP ‘high-in’ nutrition symbol for the three nutrients by food category**

| **Food categories** | | **Full or conditional exemption*** | **Saturated fat** | **Total sugar** | **Sodium** |
| --- | --- | --- | --- | --- | --- |
| RTE breakfast cereals | Puffed and uncoated | None | ≥ 10% DV | | |
|  | Puffed and coated, flaked, extruded, without fruit or nuts |  | ≥ 10% DV | | |
|  | Fruit and nut type, granola |  | ≥ 15% DV | | |
| Sliced breads | | None | ≥ 15% DV | | |
| Luncheon meats | Dried meat and poultry (e.g. parma ham, salami) | None | ≥ 10% DV | | |
|  | Luncheon meats (e.g. bologna, mortadella, ham) |  | ≥ 15% DV | | |
| Ready-to-serve soups | | None | ≥ 15% DV | | |
| Pizzas | | None | ≥ 30% DV | | |
| Frozen meals | | None | ≥ 30% DV | | |
| Granola bars | Grain-based bars and cluster with filling or partial or full coating | None | ≥ 15% DV | | |
|  | Grain-based bars and clusters without filling or coating |  | ≥ 10% DV | | |
|  | Energy and protein bars |  | ≥ 15% DV | | |
| Yogurts and dairy desserts | | Products having ≥ 5% DV calcium per serving or RA, whichever is greatest | ≥ 15% DV | | |
| Pasta sauces | | None | ≥ 15% DV | | |
| Cookies | | None | ≥ 10% DV | | |
| Sausages | | None | ≥ 15% DV | | |
| Crackers | | None | ≥ 10% DV | | |
| Salty snacks | | None | ≥ 15% DV | | |
| Processed cheeses | | Products having ≥ 5% DV calcium per serving or RA, whichever is greatest | ≥ 10% DV | | |
| Flavoured milks and plant-based beverages | | Flavoured milks that are in a refillable glass container | ≥ 15% DV | | |

*DV, Daily Value; RA, Reference Amount*

* For the food categories having a conditional exemption (i.e., yogurts and dairy desserts, and processed cheeses), it is nutrient-specific based on their ingredients. Products lose their conditional exemption for a specific nutrient when they contain an ingredient that has saturated fat, sugar or sodium other than the ingredients allowed in the *Front-of-package nutrition symbol labelling guide for industry* (Health Canada. Front-of-package nutrition symbol labelling guide for industry. Version 2. 2023).
